# Consumption of Added Sugar among Chinese Toddlers and Its Association with Picky Eating and Daily Screen Time

**DOI:** 10.3390/nu14091840

**Published:** 2022-04-28

**Authors:** Pin Li, Zhongxia Ren, Jian Zhang, Hanglian Lan, Ignatius Man-Yau Szeto, Peiyu Wang, Ai Zhao, Yumei Zhang

**Affiliations:** 1 Department of Nutrition and Food Hygiene, School of Public Health, Peking University Health Science Center, Beijing 100191, China; 2111110222@pku.edu.cn (P.L.); renzhongxia@bjmu.edu.cn (Z.R.); zhangjian92@pku.edu.cn (J.Z.); 2 Yili Maternal and Infant Nutrition Institute, Inner Mongolia Yili Industrial Group Co., Ltd., Hohhot 010110, China; lanhanglian@yili.com (H.L.); szeto@yili.com (I.M.-Y.S.); 3 Inner Mongolia Dairy Technology Research Institute Co., Ltd., Hohhot 010110, China; 4 Department of Social Science and Health Education, School of Public Health, Peking University Health Science Center, Beijing 100191, China; wpeiyu@bjmu.edu.cn; 5 Vanke School of Public Health, Tsinghua University, Beijing 100084, China; 6 Beijing Key Laboratory of Toxicological Research and Risk Assessment for Food Safety, School of Public Health, Peking University, Beijing 100022, China

**Keywords:** key toddlers, added sugar, picky eating, screen time, zero-inflated negative binomial model

## Abstract

Objectives: This study aimed to examine the relationship between daily screen time, picky eating, and consumption frequency of sugared foods and sugar-sweetened beverages (SSBs). Methods: The research data came from the Young Investigation (YI) study conducted in 10 cities in China. This study used sociodemographic information, feeding behavior, picky eating reported by parents, and the consumption frequency of sugared foods and SSBs of 879 toddlers aged 1–3 years. The relationship between daily screen time and picky eating behavior was assessed using logistic regression. The zero-inflated negative binomial (ZINB) model was used to fit the consumption frequencies of sugared foods and SSBs. Results: In all, 13.1% (*n* = 115) of toddlers did not have sugared foods 1 month before the survey, while 73.3% (*n* = 644) of toddlers did not have SSBs 1 month before the survey. The consumption rate of sugared foods was relatively higher than SSBs (χ^2^ = 661.25, *p* < 0.001). After adjusting for social demographic information, no relationship was found between daily screen time and picky eating (OR = 1.437; 95% CI: 0.990,2.092). The ZINB model showed that, among children who ate sugared foods, children who were picky eaters ate them more often (IRR = 1.133; 95% CI: 1.095,1.172), but no association was found between picky eating and the chance of avoiding sugared foods (OR = 0.949; 95% CI: 0.613,1.471). Children who were picky eaters were less likely not to drink SSBs (OR = 0.664; 95% CI: 0.478,0.921). However, among children who consumed SSBs, picky eaters drank them less frequently (IRR = 0.599; 95% CI: 0.552,0.650). Children with a screen time of no less than 1 h/d ate sugared foods more frequently (IRR = 1.383; 95% CI: 1.164,1.644), and they were less likely to avoid sugared foods (OR = 0.223; 95% CI: 0.085,0.587). The longer the screen time per day was, the less likely children did not have SSBs (<1 h/d: OR = 0.272; 95% CI: 0.130, 0.569; ≥1 h/d: OR = 0.136; 95% CI: 0.057, 0.328). Conclusions: The consumption rate of sugared foods was higher than that of SSBs. Picky eating and daily screen time were related to the consumption frequency of added sugar among Chinese toddlers aged 1–3 years. Picky eaters consumed sugared foods more frequently and were more likely to drink SSBs. Children whose daily screen time reached 1 h/d were more likely to eat sugared foods and drink SSBs.

## 1. Introduction

The triple burden of malnutrition (undernutrition, hidden hunger, and overweight) threatens the growth and development of children. The phenomenon of the undernutrition of infants has decreased significantly, and the incidence of overweight and obesity has increased rapidly [1], indicating that malnutrition has become the main nutritional problem of children all over the world. Toddlerhood is a key period for the establishment of eating behaviors and food preferences [2]. The nutritional status of infants and toddlers may have a long-term impact on health [3]. A healthy diet can effectively reduce the risk of malnutrition [4].

Studies on the psychological and physical traits of children have confirmed their preference for sweetness [5]. The early introduction of sweetened foods has been observed in various populations. Observational studies have also demonstrated that there is a high proportion of infants eating unhealthy foods and drinks, such as candy and juice [6]. A multicenter study conducted in Europe found that more than 95% of infants began to eat sweets by the age of 9 months [7]. The National Health and Nutrition Examination (NHANES) data from 2009–2012 showed that the energy percent contributed by added sugar was about 14.3% for children aged 2–8 years, exceeding the 10% guideline [8]. The intake of sugar-sweetened beverages (SSBs) has a strong correlation between 2 and 5 years of age [9]. Therefore, intervention measures to reduce the amount of added sugar should be started as soon as possible.

Sugared foods and SSBs are the main sources of added sugar [8,9]. The intake of sugared foods is one of the major reasons of developing overweight and obesity [10,11]. Chinese studies further identified that SSBs are independently associated with a high risk of abdominal obesity [12].

The quality of the diet of children is related to a variety of factors, such as sex, region, education levels of parents, socioeconomic status (SES), physical activity, and sleep duration [13,14]. What is more, children’s screen time has increased significantly during the COVID-19 pandemic [15]. Screen time was found to be associated with increased intake frequencies of unhealthy foods [16,17,18]. In addition to the effects of increasing screen time, picky eating is also one reason for the poor dietary quality of toddlers [19,20]. Picky eating is an umbrella term describing the rejection of one or more food items, which might be accompanied by strong food preferences [21,22]. Previous studies have found that children with high picky eating levels have insufficient intakes of vitamins, minerals, and dietary fiber [23] but a higher intake of trans fatty acids [24]. In addition, it has been reported that children who use a screen for longer times were more likely to be picky about food [25], so there might be an interaction between picky eating, daily screen time, and diet quality.

Previous studies have mostly explored the impact of screen time and picky eating on diets separately, but few studies have examined the relationship between screen time, picky eating, and a sugary diet. This study aimed to explore the associations among screen time, picky eating, and intake of foods and drinks with added sugar in 10 cities in China.

## 2. Materials and Methods

### 2.1. Subjects

This study was based on the Young Investigation (YI) study, which was a cross-sectional survey focused on the nutrition and health of pregnant women, mother–infant pairs, and toddlers (aged 1–3 years). The YI study was conducted in 10 cities in China from 2019 to 2020. The 10 cities surveyed included two first-tier cities (Beijing and Guangzhou); three new first-tier cities (Suzhou, Chengdu, and Shenyang); three second-tier cities (Ningbo, Nanchang, and Lanzhou); one third-tier city (Hohhot); and one fourth-tier city (Xuchang). A general hospital or maternal and child health hospital was selected in each city to recruit the respondents. The inclusion criteria of toddlers included the following: (1) singleton, (2) term birth (gestational age ≥ 37 weeks), and (3) 12–35.9 months old. The exclusion criteria were as follows: (1) physical disabilities; (2) recently experienced infectious, metabolic, mental, and other major diseases; or (3) the guardian could not answer the questions. Finally, at least 90 children were selected in each city for the investigation. The original investigation included 924 individuals. After excluding those missing important information (sociodemographic information, outdoor activities, sleep duration, picky eating behavior, daily screen time, and consumption frequency of sugared foods and SSBs), the data of 879 toddlers were finally used.

### 2.2. Data Collection

With a face-to-face survey, the uniformly trained investigators asked the main caregivers to convey the sociodemographic information (age, sex, delivery mode, parity, family income, mother’s education level, mother’s working state, place of residence, etc.); exact daily time of outdoor activities and sleep duration (daytime and nighttime); picky eating behavior; and daily screen time of their toddlers based on a self-developed questionnaire. For the evaluation of the children’s daily screen time, the investigator asked the main caregivers of the toddler in detail about the specific time using the screen every day. The daily screen time was the sum of the time children spend watching TV or using computers, tablets, mobile phones, and other electronic products a day. Picky eating behavior was self-reported by the main feeders of the toddlers, which was mainly manifested in their rejection of eating one or more kinds of food, including milk, beans, cereals, vegetables, fruits, meat, etc. The intake frequency of sugared foods and SSBs over the past month were assessed using a food frequency questionnaire (FFQ). The foods with added sugar included cake, candy, chocolate, etc. SSBs included water with added sugar, flavored milk drinks, carbonated drinks, etc. Food and beverages that contained different kinds of sweeteners (besides sugar) were not included. Freshly squeezed fruit and vegetable juices were not counted as SSBs in this study.

### 2.3. Statistical Analysis

All of the statistical analyses were performed using R software (version 4.1.1). Descriptive statistics were presented as numbers (percentages). Children’s screen time was converted to categorical variables (0 h/d; <1 h/d; ≥1 h/d). In the univariate analysis, the monthly consumption frequency of sugared foods and SSBs was divided into three levels, and the chi-square test was used to analyze the differences in the lifestyle and sociodemographic characteristics between different groups. Taking those children whose screen time was 0 h/d as the reference group, the associations between different screen times and picky eating groups were analyzed by multivariate logistic regression after adjusting for the age, sex, family income, mother’s educational level, place of residence, outdoor activity time, and daytime and nighttime durations of the toddlers (the independent variable was daily screen time, and the dependent variable was picky eating behavior).

The distribution of the consumption frequency of foods and drinks with added sugar was non-normal count data, and the variance was greater than the mean presented. In the meantime, there were excess zero counts in the data (13.3% and 73.3% in the consumption frequency of sugared foods and SSBs, respectively), so we considered the consumption frequency as an over-dispersed and zero-inflated count variable. Therefore, taking picky eating and daily screen time as independent variables and the sugar-containing food and SSB consumption frequency as dependent variables, the zero-inflated negative binomial (ZINB) model was adopted to assess the associations among picky eating behavior, daily screen time, and the consumption frequency of sugared foods and SSBs, including intake frequency and the chance of not having any sugared food/drinks in the past month, such as a “sugar-free diet”.

As described by Cheung [26], the ZINB model satisfies the over-discretization of the dependent variable data structure. Its application assumes that the data variance is greater than the mean, which is in line with the characteristics of the data structure in this paper. In addition, the ZINB model is more suitable to solve the problem of too many zero counts in the data. ZINB modeling generated two separate models: a logit model for the “sugar-free diet” samples predicting the chance of being in the “sugar-free” group and a negative binomial model analyzing the intake frequency for those children who had sugared foods/drinks. The model was sequentially adjusted for the demographic features (age, sex, family income, mother’s education level, and place of residence), then additionally for lifestyle behaviors (outdoor time, daytime sleep, and nighttime sleep).

To confirm the results of the ZINB model, the standard negative binomial (NB) regression model was also used with the same adjustment of variables. The ZINB and NB models were conducted using the “pscl” and “MASS” packages, respectively. The significance level for all the statistical tests was set at 0.05.

## 3. Results

### 3.1. Factors Associated with Consumption Frequency of Added Sugar

The consumption rate of sugared foods was 86.9%, which was higher than the consumption rate of SSBs (χ2 = 661.25, *p* < 0.001). The sociodemographic characteristics, feeding-related characteristics, and daily screen times of children with different levels of monthly consumption frequencies of added sugar are summarized in Table 1. The frequency of consumption of sugared foods and beverages in children over 2 years old was higher. Additionally, a lower maternal educational level and picky eating were associated with a higher consumption frequency of drinks with added sugar. Children with longer daily screen times also consumed both drinks and foods with added sugar more often. However, the differences in sex, delivery mode, parity, mother’s working state, family income, and the primary caregivers between the groups were not significant.

### 3.2. Association between Picky Eating and Daily Screen Time

The association between self-reported picky eating behavior from parents and screen time is shown in Table 2. Although, in the crude model without any adjustment (model 1) and the model only adjusting for age and sex (model 2), children who used a screen had a higher incidence of picky eating, an association between daily screen time and picky eating behaviors of children was not found after adjusting for the sociodemographic information (model 3) and lifestyle factors (model 4).

### 3.3. Association between Picky Eating and Consumption Frequency of Added Sugar

The results of the ZINB model are shown in Table 3, including the relationship between picky eating and the consumption frequency of added sugar, as well as the possibility of not eating a sugary diet, respectively. Among children who had a sugary diet in the negative binomial model part, we found that children who were picky eaters had a higher consumption frequency of foods with added sugar but a lower frequency of the consumption of SSBs. In the logit model, indicating the chance of being “sugar-free”, no association was found between picky eating and sugared foods, but children who were picky eaters were less likely not to drink SSBs. On the other hand, when taking into consideration only the standard negative binomial regression model, picky eating behavior was not associated with the frequency of the consumption of foods and drinks with added sugar.

### 3.4. Association between Daily Screen Time and Consumption Frequency of Added Sugar

Table 4 presents the results of the relationship between daily screen time and the eating behavior of added sugar using the ZINB model and standard negative binomial regression.

Firstly, the analysis of sugared foods found that children whose screen time reached 1 h/d had a higher consumption frequency and lower possibility of not eating sugared foods than those whose screen time was 0 h/d. However, the added sugar consumption behavior of children with <1 h of screen time per day was not different from that of children in the reference group. The results of the standard negative binomial regression analysis also confirmed that the consumption frequency of sugared foods of children with a screen time of 1 h/d was significantly higher than that of children who did not use screens.

The negative binomial model of the ZINB model of SSBs indicated that children whose screen time was <1 h/d drank SSBs less frequently. On the other hand, the logit model of the ZINB model showed that children who used screens were less likely not to have SSBs. Additionally, the results of the standard negative binomial regression test were similar to those with sugared foods—namely, children whose screen time was ≥1 h/d drank SSBs more frequently.

## 4. Discussion

**Figure 1:**
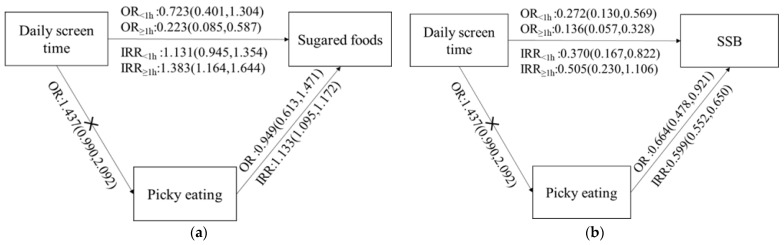
(**a**) Relationship between daily screen time, picky eating, and the consumption frequency of sugared foods. (**b**) Relationship between daily screen time, picky eating, and the consumption frequency of SSBs. IRR, incidence rate ratio; OR, odds ratio; SSBs, sugar-sweetened beverages.

This study assessed the relationship between daily screen time, picky eating, and a sugary diet. We did not find a correlation between the total daily screen time and picky eating, and the results showed that both picky eating and screen time were related to children’s consumption of sugared foods and SSBs (summarized in Figure 1).

### 4.1. Sugared Foods and Beverage Consumption and Its Associated Factors

In our study, the consumption rate of sugared foods was relatively higher than that of SSBs. The intake rates of sugared foods and SSBs were lower than previous studies. According to a cross-sectional survey in The Netherlands, 20.2% and 16.5% of 6-month-old infants consumed sugared foods and SSBs every day, respectively [27]. In the United States, more than a quarter of children under the age of 2 drank SSBs during their dietary recall [6]. The consumption rate of young children in developing countries has also reached a high level—60.0% of children aged 24–35 months in Indonesia ate snacks three or more times a day [28]. Previous studies have reported that an added sugar consumption increases significantly with age [29]. This study only focused on toddlers aged 1–3 years and found that the sugared foods intake was higher in children in the 24–36-month-old age group, and it might be increased alongside children’s growth. This also suggested that Chinese parents might begin to control their children’s intake of added sugar as early as possible. Additionally, an association between the mother’s educational level and added sugar was not observed in this study, but some researchers pointed out that the mother’s educational level is related to a sugary diet in children [28,30]. In addition, the participants in our study were recruited from cities, and most of their mothers were well-educated.

### 4.2. Association of Sugared Foods and SSBs with Picky Eating

Picky eating behavior has different effects on the consumption of sugared foods and SSBs. Children who were picky eaters consumed sugared foods more frequently. Although children who are picky eaters are more likely to consume SSBs, their consumption frequency was lower, which is contrary to previous studies. A cohort study carried out in the United Kingdom found that, among older children who were picky eaters, the intake of sugared foods and SSBs was higher [22].

### 4.3. Association of Sugared Foods and SSBs with Screen Time

Our study proved that children whose screen time reached 1 h/d were more likely to eat sugared foods and SSBs, and sugared foods were eaten more frequently. However, the negative binomial part of the ZINB model showed that the consumption frequency of SSBs of children with <1 h/d of screen time per day was lower than those who did not use screens.

### 4.4. Association among Screen Time, Picky Eating, and Intake of Sugared Foods and SSBs

The reason for the inconsistent results of the consumption frequency of SSBs might be because those children who drank SSBs were only a small part of the respondents, which is bound to have an impact on the results. The results of this study are basically consistent with previous studies. A cross-sectional study on Chinese children and adolescents showed that those who were non-consumers of SSBs were less likely to have ≥2 h/d of screen time [31]. Similar research on Greek schoolchildren aged 8–17 years also pointed out that a longer screen time increased the possibility of unhealthy eating habits, such as skipping breakfast, often eating fast food, and often eating sweets [32]. A prospective European study found that the odds ratio of having increased consumption of SSBs was 1.19 for each hour per day watching TV [33].

The WHO suggested that screen time is not recommended for infants and children less than 2 years of age, and for those aged 2 to 3 years, screen time should be no more than 1 h/d; less is better [34]. In addition to the effect on a sugary diet, screen exposure is associated with the increasing prevalence of overweight and obesity [33,35,36]. Meanwhile, the overuse of digital media is harmful for the development of physical and cognitive abilities, sleeping, and the emotions of children [35,37,38]. Randomized controlled trials of reducing screen time in a community reduced the weight gains of children [39].

Children’s picky eating behavior, screen use time, and dessert intake are closely related to the living environment provided by their parents [40,41]. Research on Chinese teenagers found that the attitudes of parents toward sweets will affect the intake of sweets by children by affecting the availability of sweets in the family [42]. Parental and family-based interventions that support the development of healthy eating rules for children have the potential to improve their children’s diet quality, such as changing feeding methods or helping infants to identify unhealthy foods [43,44].

The effect of screen time on a diet may be related to advertising [45,46]. Children are exposed to foods and beverages in the advertisements they watch, as well as during children-specific programs. The proportion of ultra-processed foods in a diet was positively correlated with the intake of leisure foods and added sugar, and this was particularly evident among younger children [28,47].

### 4.5. Strengths and Limitations

There were several strengths in this study. The participants were recruited from 10 cities in China, and the relationships among sugared food intake, picky eating, and screen time were fully evaluated. However, as with a cross-sectional design, recall bias may exist, and we could only detect associations but were not able to infer or establish any causality. For the methodology, although we tried our best to ensure that the child’s primary caregiver answered all questions in the survey, the evaluations of picky eating behavior and daily screen time were based on self-reported questions instead of standardized tools, which may have led to a misclassification bias. In addition, although we adjusted for confounders that may have influenced the results, residual confounders may have still existed. Further retrospective studies are still needed to examine the effects of screen exposure on the diets and picky eating behaviors of toddlers.

## 5. Conclusions

The consumption rate of sugared foods was higher than that of SSBs. Picky eating and daily screen time were related to the consumption frequency of added sugar among Chinese toddlers. Picky eaters consumed sugared foods more frequently and were more likely to drink SSBs. Children whose screen times reached ≥1 h/d were more likely to eat sugared foods and SSBs.

This study highlighted the importance of early intervention on reducing the consumption frequency of sugared foods. The toddler period is a critical window for building healthy eating behaviors. A comprehensive strategy, including reducing screen times and avoiding picky eating behaviors, may benefit by reducing the consumption frequency of sugared foods and bring about a lifelong health effect.

## Figures and Tables

**Table 1:** Monthly consumption frequency of added sugar, *n* (%).

Variables		N (%)	Monthly Sugar-Added Food Consumption Frequency				Monthly SSBs Consumption Frequency			
			0	1~15	16~	*p*-Value	0	1~4	5~	*p*-Value
Age	1~	483 (54.9)	82 (71.3)	229 (54.4)	172 (50.1)	**<0.001**	393 (61.0)	47 (38.8)	43 (37.7)	**<0.001**
	2~	396 (45.1)	33 (28.7)	192 (45.6)	171 (49.9)		251 (39.0)	74 (61.2)	71 (62.3)	
Sex	female	428 (48.7)	59 (51.3)	190 (45.1)	179 (52.2)	0.127	304 (47.2)	63 (52.1)	61 (53.5)	0.336
	male	451 (51.3)	56 (48.7)	231 (54.9)	164 (47.8)		340 (52.8)	58 (47.9)	53 (46.5)	
Delivery mode	vaginal	493 (56.1)	64 (55.7)	233 (55.3)	196 (57.1)	0.879	352 (54.7)	71 (58.7)	70 (61.4)	0.338
	cesarean	386 (43.9)	51 (44.3)	188 (44.7)	147 (42.9)		292 (45.3)	50 (41.3)	44 (38.6)	
Parity	1	545 (62.0)	75 (65.2)	253 (60.1)	217 (63.3)	0.500	392 (60.9)	78 (64.5)	75 (65.8)	0.508
	>1	334 (38.0)	40 (34.8)	168 (39.9)	126 (36.7)		252 (39.1)	43 (35.5)	39 (34.2)	
Mother’s	working	561 (63.8)	71 (61.7)	263 (62.5)	227 (66.2)	0.503	420 (65.2)	74 (61.2)	67 (58.8)	0.337
working state	not working	318 (36.2)	44 (38.3)	158 (37.5)	116 (33.8)		224 (34.8)	47 (38.8)	47 (41.2)	
Mother’s	0~9	112 (12.7)	12 (10.4)	51 (12.1)	49 (14.3)	0.409	70 (10.9)	21 (17.4)	21 (18.4)	**0.013**
educational level	12	138 (15.7)	17 (14.8)	75 (17.8)	46 (13.4)		95 (14.8)	26 (21.5)	17 (14.9)	
(years)	13~	629 (71.6)	86 (74.8)	295 (70.1)	248 (72.3)		479 (74.4)	74 (61.2)	76 (66.7)	
Per capita	0~	402 (45.7)	47 (40.9)	201 (47.7)	154 (44.9)	0.333	290 (45.0)	62 (51.2)	50 (43.9)	0.770
monthly income	5000~	315 (35.8)	44 (38.3)	138 (32.8)	133 (38.8)		233 (36.2)	39 (32.2)	43 (37.7)	
(RMB: yuan)	10,000~	162 (18.4)	24 (20.9)	82 (19.5)	56 (16.3)		121 (18.8)	20 (16.5)	21 (18.4)	
The primary	parents	472 (53.7)	64 (55.7)	230 (54.6)	178 (51.9)	0.680	332 (51.6)	69 (57.0)	71 (62.3)	0.078
caregivers	others	407 (46.3)	51 (44.3)	191 (45.4)	165 (48.1)		312 (48.4)	52 (43.0)	43 (37.7)	
Picky eating	without	565 (64.3)	78 (67.8)	273 (64.8)	214 (62.4)	0.543	437 (67.9)	59 (48.8)	69 (60.5)	**<0.001**
	with	314 (35.7)	37 (32.2)	148 (35.2)	129 (37.6)		207 (32.1)	62 (51.2)	45 (39.5)	
Daily	0	275 (31.3)	55 (47.8)	138 (32.8)	82 (23.9)	**<0.001**	243 (37.7)	15 (12.4)	17 (14.9)	**<0.001**
screen time	<1	242 (27.5)	36 (31.3)	111 (26.4)	95 (27.7)		182 (28.3)	37 (30.6)	23 (20.2)	
(h/d)	≥1	362 (41.2)	24 (20.9)	172 (40.9)	166 (48.4)		219 (34.0)	69 (57.0)	74 (64.9)	
Total		879 (100.0)	115 (13.1)	421 (47.9)	343 (39.0)		644 (73.3)	121(13.8)	114(13.0)	

Bold text represents a statistically significant difference (*p* < 0.05); *p*-values were obtained from the chi-square test. SSBs, sugar-sweetened beverages.

**Table 2:** Associations between picky eating and daily screen time.

Daily Screen Time	OR	95% CI	*p*-Value
Model 1 ^a^			
0 h	1(Ref)		
<1 h	1.158	(0.795, 1.687)	0.445
≥1 h	1.922	(1.380, 2.691)	<0.001
Model 2 ^b^			
0 h	1(Ref)		
<1 h	1.017	(0.690, 1.497)	0.933
≥1 h	1.555	(1.084, 2.237)	0.017
Model 3 ^c^			
0 h	1(Ref)		
<1 h	1.043	(0.702, 1.547)	0.835
≥1 h	1.432	(0.987, 2.084)	0.059
Model 4 ^d^			
0 h	1(Ref)		
<1 h	1.040	(0.700, 1.544)	0.845
≥1 h	1.437	(0.990, 2.092)	0.057

Daily screen time was represented by three levels (0 h, <1 h, and ≥1 h). The reference category is 0 h. Picky eating behavior is the dependent variable. OR, odds ratio. ^a^ unadjusted model; ^b^ adjusted for toddlers’ age and sex; ^c^ adjusted for toddlers’ age, sex, family income, mother’s educational level, and place of residence; and ^d^ adjusted for toddlers’ age, sex, family income, mother’s educational level, place of residence, outdoor activity time, and sleep duration.

**Table 3:** Associations between picky eating and the consumption frequency of products with added sugar.

	Monthly Sugar-Added Food Consumption Frequency			Monthly SSBs Consumption Frequency		
**Picky Eating**	**Zero-Inflated NB Model**		**NB Regression Model**	**Zero-Inflated NB Model**		**NB Regression Model**
	**Negative Binomial Part**	**Zero-Inflated Part**	**IRR (95% CI)**	**Negative Binomial Part**	**Zero-Inflated Part**	**IRR (95% CI)**
	**IRR (95% CI)**	**OR (95% CI)**		**IRR (95% CI)**	**OR (95% CI)**	
Model 1 ^a^						
without	1(Ref)			1(Ref)		
with	1.169(1.017, 1.343) *	0.839(0.484, 1.456)	1.189(1.015, 1.397) *	0.571(0.371, 0.881) *	0.348(0.172, 0.705) **	0.957(0.601, 1.555)
Model 2 ^b^						
without	1(Ref)			1(Ref)		
with	1.136(1.098, 1.175) ***	0.953(0.616, 1.475)	1.133(0.963, 1.334)	0.609(0.561, 0.660) ***	0.681(0.492, 0.941) *	0.928(0.558, 1.563)
Model 3 ^c^						
without	1(Ref)			1(Ref)		
with	1.133(1.095, 1.172) ***	0.949(0.613, 1.471)	1.132(0.962, 1.334)	0.599(0.552, 0.650) ***	0.664(0.478, 0.921) *	0.966(0.586, 1.608)

The monthly consumption frequency of sugar-added foods and SSBs is the dependent variable (counting data). IRR, incidence rate ratio; OR, odds ratio. SSBs, sugar-sweetened beverages. NB, negative binomial. ^a^ unadjusted model; ^b^ adjusted for toddlers’ age, sex, family income, mother’s educational level, and place of residence; and ^c^ adjusted for toddlers’ age, sex, family income, mother’s educational level, place of residence, outdoor activity time, and sleep duration. * *p* < 0.05, ** *p* < 0.01, and *** *p* < 0.001.

**Table 4:** Associations between daily screen time and the consumption frequency of products with added sugar.

Daily Screen Time	Monthly Sugar-Added Food Consumption Frequency			Monthly SSBs Consumption Frequency		
	Zero-Inflated NB Model		NB Regression Model IRR (95% CI)	Zero-Inflated NB Model		NB Regression Model IRR (95% CI)
	Negative Binomial Part IRR (95% CI)	Zero-Inflated Part OR (95% CI)		Negative Binomial Part IRR (95% CI)	Zero-Inflated Part OR (95% CI)	
Model 1 ^a^						
0 h	1(Ref)			1(Ref)		
<1 h	1.104(0.923, 1.321)	0.664(0.374, 1.180)	1.170(0.959, 1.428)	0.483(0.233, 1.002)	0.263(0.131, 0.526) ***	1.184(0.66, 2.137)
≥1 h	1.316(1.122, 1.544) ***	0.199(0.088, 0.454) ***	1.519(1.268, 1.818) ***	0.787(0.408, 1.516)	0.107(0.040, 0.282) ***	2.785(1.63, 4.702) ***
Model 2 ^b^						
0 h	1(Ref)			1(Ref)		
<1 h	1.135(0.946,1.361)	0.720(0.393,1.317)	1.181(0.965,1.445)	0.411(0.188,0.899) *	0.283(0.135,0.596) ***	1.510(0.789,2.910)
≥1 h	1.366(1.147,1.627) ***	0.172(0.043,0.695) *	1.565(1.288,1.901) ***	0.631(0.299,1.333)	0.139(0.061,0.320) ***	3.096(1.672,5.690) ***
Model 3 ^c^						
0 h	1(Ref)			1(Ref)		
<1 h	1.131(0.945,1.354)	0.723(0.401,1.304)	1.182(0.966,1.448)	0.370(0.167,0.822) *	0.272(0.130,0.569) ***	1.528(0.795,2.950)
≥1 h	1.383(1.164,1.644) ***	0.223(0.085,0.587) **	1.577(1.296,1.916) ***	0.505(0.230,1.106)	0.136(0.057,0.328) ***	2.637(1.396,4.963) ***

The monthly consumption frequency of sugar-added foods and SSBs is the dependent variable (counting data). IRR, incidence rate ratio; OR, odds ratio. SSBs, sugar-sweetened beverages. NB, negative binomial ^a^ unadjusted model; ^b^ adjusted for toddlers’ age, sex, family income, mother’s educational level, and place of residence; and ^c^ adjusted for toddlers’ age, sex, family income, mother’s educational level, place of residence, outdoor activity time, and sleep duration. * *p* < 0.05, ** *p* < 0.01, and *** *p* < 0.001.
